# Pharmacologic Inhibition of Histone Deacetylase 6 Prevents the Progression of Chlorhexidine Gluconate-Induced Peritoneal Fibrosis by Blockade of M2 Macrophage Polarization

**DOI:** 10.3389/fimmu.2022.899140

**Published:** 2022-06-15

**Authors:** Yingfeng Shi, Jinqing Li, Hui Chen, Yan Hu, Lunxian Tang, Xun Zhou, Min Tao, Zexin Lv, Si Chen, Andong Qiu, Na Liu

**Affiliations:** ^1^ Department of Nephrology, Shanghai East Hospital, Tongji University School of Medicine, Shanghai, China; ^2^ Emergency Department of Critical Care Medicine, Shanghai East Hospital, Tongji University School of Medicine, Shanghai, China; ^3^ School of Life Science and Technology, Advanced Institute of Translational Medicine, Tongji University, Shanghai, China

**Keywords:** peritoneal dialysis, peritoneal fibrosis, histone deacetylase 6, macrophage polarization, tubastatin A

## Abstract

Peritoneal fibrosis contributes to ultrafiltration failure in peritoneal dialysis (PD) patients and thus restricts the wide application of PD in clinic. Recently we have demonstrated that histone deacetylase 6 (HDAC6) is critically implicated in high glucose peritoneal dialysis fluid (HG-PDF) induced peritoneal fibrosis, however, the precise mechanisms of HDAC6 in peritoneal fibrosis have not been elucidated. Here, we focused on the role and mechanisms of HDAC6 in chlorhexidine gluconate (CG) induced peritoneal fibrosis and discussed the mechanisms involved. We found Tubastatin A (TA), a selective inhibitor of HDAC6, significantly prevented the progression of peritoneal fibrosis, as characterized by reduction of epithelial-mesenchymal transition (EMT) and extracellular matrix (ECM) protein deposition. Inhibition of HDAC6 remarkably suppressed the expression of matrix metalloproteinases-2 (MMP2) and MMP-9. Administration of TA also increased the expression of acetylation Histone H3 and acetylation α-tubulin. Moreover, our results revealed that blockade of HDAC6 inhibited alternatively M2 macrophages polarization by suppressing the activation of TGF-β/Smad3, PI3K/AKT, and STAT3, STAT6 pathways. To give a better understanding of the mechanisms, we further established two cell injured models in Raw264.7 cells by using IL-4 and HG-PDF. Our *in vitro* experiments illustrated that both IL-4 and HG-PDF could induce M2 macrophage polarization, as demonstrated by upregulation of CD163 and Arginase-1. Inhibition of HDAC6 by TA significantly abrogated M2 macrophage polarization dose-dependently by suppressing TGF-β/Smad, IL4/STAT6, and PI3K/AKT signaling pathways. Collectively, our study revealed that blockade of HDAC6 by TA could suppress the progression of CG-induced peritoneal fibrosis by blockade of M2 macrophage polarization. Thus, HDAC6 may be a promising target in peritoneal fibrosis treatment.

## Introduction

It has been reported the prevalence of end-stage kidney diseases (ESKD) is continually increasing (1), and peritoneal dialysis (PD) is an alternative way of renal replacement therapy for patients with ESKD (2). Despite the similar outcome with hemodialysis (3), PD is still not the first choice for most patients with chronic kidney disease stage 5. Only about 13% dialysis patients in European and about 10% dialysis patients in the USA receive PD (4). The clinical application of PD as a renal replacement therapy is limited by the occurrence of ultrafiltration failure (UFF), which can result in withdrawal from dialysis. Some studies revealed the prevalence of UFF in long-term PD patients may exceed 30% (5). The decline of the ultrafiltration capacity (the ability to remove the excess water and metabolic waste products) is ascribed to many complicated reasons. Among the causes of UFF, peritoneal fibrosis must not be overlooked.

The mechanisms by which regulate peritoneal fibrosis have been a hot point in PD field for a long time, including the chronic peritoneal inflammation state (6), angiogenesis, epithelial-to-mesenchymal (EMT) and so on (7, 8). The interplay between these processes plays a great role to promote the pathophysiology process of peritoneal fibrosis. Recently, inflammation has become increasingly important in the process of peritoneal fibrosis, and inflammatory microenvironment can accelerate the development of angiogenesis and EMT (8, 9). Several inflammatory pathways are involved in the peritoneal fibrosis such as NOD-like receptor protein 3 (NLRP3), Toll-like receptor (TLR), NF-κB, interleukin (IL)-1β, IL-6, IL-17, and other cytokines (8). Moreover, immune cell infiltration is also reported to participate in peritoneal fibrosis, which includes the alternative activation of macrophages (M2) (10, 11).

Macrophages are immune cells that can be polarized into different subtypes by various stimuli and microenvironment (12). Generally, the classically activated macrophages (M1) can be induced by lipopolysaccharide (LPS) and interferon-γ (IFN-γ), while the alternative activation of macrophages (M2) can be induced by IL-4 and IL-13 (12–14). It has been demonstrated that M2 macrophage polarization is related to fibrotic remodeling and tissue repair of multiple internal organs, including heart, kidney, liver, gastrointestinal tract and lung (15, 16). Recent studies identified that microenvironment stimulates macrophage M2 polarization mostly through activation of TGF-β/Smad, IL4/STAT6, PI3K/AKT signaling pathways (17–20). We used to find CD68-positive macrophage cells in the thickened sub-mesothelial area in our murine peritoneal fibrosis model (21). Meanwhile, M2 macrophages are considered to participate in peritoneal fibrosis and the depletion of M2 macrophages in peritoneum could mitigate the peritoneal fibrosis in the previous research (22). However, the underlying mechanisms to regulate M2 macrophage polarization during peritoneal fibrosis are still not clear.

On the other hand, several studies have shown the critical role of epigenetic regulation in fibrotic diseases in recent years. Acetylation is one of the ways of post-translational modifications, and the histone deacetylase [HDAC; also named as lysine deacetylases (KDACs)] family is a group of key factors regulating the acetylation in a series of physiological and pathological activities (23, 24). HDAC6, which is mainly located in the cytoplasm, is a unique member of the HDAC family and has effects on both histone and nonhistone acetylation. The main nonhistone substrates of HDAC6 include α-tubulin, cortactin and HSP90 (25). In addition to its involvement in tumor proliferation, invasion and metastasis (25), HDAC6 also plays an important role in cardiac fibrosis (26), renal fibrosis (27), peritoneal fibrosis and many other fibrotic diseases. Except for HDAC6, another subtype of HDAC family, HDAC1 from class I has also been confirmed to be associated with peritoneal fibrosis (28). HDAC1 was reported to induce mesothelial to mesenchymal transition (MMT)-related marker gene expression in mesothelial cells isolated from effluent of PD patients. Thus, this research pinpointed a role for HDAC1 as a new player in the regulation of peritoneal fibrosis (28). In our previous study, we found an elevated expression of HDAC6 in peritoneum and dialysis effluent from PD patients (29). Moreover, we demonstrated that HDAC6 was indispensable in interleukin-6 induced EMT, proliferation and migration of peritoneal mesothelial cells (30). However, the link between HDAC6 and M2 polarization in PF remains unclear.

In this study, we explored the role of HDAC6 in M2 macrophage polarization in a chlorhexidine gluconate (CG)-induced peritoneal fibrosis model by using a selective HDAC6 inhibitor Tubastatin A (TA). We further established two cell injured models in Raw264.7 cells by using IL-4 and high glucose peritoneal dialysis fluid (HG-PDF), and explored the relevant regulatory mechanisms, including TGF-β/Smad, IL4/STAT6, and PI3K/AKT signaling pathways. This study will further clarify the role and mechanism of HDAC6 in M2 macrophage polarization, and recommend HDAC6 as a potential target for therapy of peritoneal fibrosis in the future.

## Materials and Methods

### Antibodies and Reagents

Tubastatin A was purchased from Selleckchem (Houston, TX, United States). Antibodies to HDAC6 (#7612), Acetyl Histone H3 (Lys9) (#9649), Histone H3 (#9717), Acetyl α-Tubulin (Lys40) (#5335), α-Tubulin (#3873), Smad3 (#9523), p-Smad3 (#9520), TAK1 (#5206), p-TAK1 (#9339), Snail (#3879), PI3K (#4257), p-PI3K (#17366), AKT (#4691), p-AKT (#4060), STAT3 (#9139), p-STAT3 (#9138), CTGF (#86641), E-cadherin (#14472), STAT6 (#5397) and p-STAT6 (#56554) were purchased from Cell Signaling Technology (Danvers, MA, United States). Antibodies to Fibronectin (ab2413), MMP2 (ab37150), MMP9 (ab38898) were purchased from Abcam (Cambridge, MA). Antibody to Twist (A3237) was purchased from ABclonal (Wuhan, China). Antibodies to GAPDH (sc-32233), Collagen I (A2) (sc-28654), CD68 (sc-20060), TGFβRI (sc-399) were purchased from Santa Cruz Biotechnology (San Diego, CA, United States). Antibodies to Arginase-1 (GB11285) and CD163 (GB11340) were purchased from Servicebio (Wuhan, China). IL-4 protein was purchased from R&D Systems (Minneapolis, MN, United States). Peritoneal dialysate was purchased from Baxter Healthcare (Guangzhou, China). Antibody to α-SMA (A2547), chlorhexidine gluconate (C9394) and all other chemicals were obtained from Sigma-Aldrich (St. Louis, MO, United States).

### Animal Model and Experimental Design

Animal experiments were reviewed and approved by the Institutional Animal Care and Use Committee at Tongji University (Shanghai, RP China). C57/black mice (provided by Shanghai Super-B&K Laboratory Animal Corp. Ltd, Shanghai, PR China) that weighed 20-25g were maintained in a pathogen-free facility under a 12 h light-dark cycle with abundant food and water supplied. All animal work was performed in Tongji University school of medicine. The mouse model of PF was established by intraperitoneal injection of 0.1% chlorhexidine gluconate (CG) (10 ml/kg) dissolved in saline every other day for 21 days as previously described (21). On the other hand, several mice in TA treatment group were injected intraperitoneally with a single dose of TA (70 mg/kg) in DMSO every day to investigate the therapeutic effects (31). Mice were randomly divided into four groups with 6 mice per group: (1) sham: mice injected with an equivalent amount of saline intraperitoneally and DMSO; (2) sham + TA: mice injected with an equivalent amount of saline intraperitoneally and 70 mg/kg TA; (3) CG: mice injected with 0.1% CG intraperitoneally and an equivalent amount of DMSO; (4) CG + TA: mice injected with 0.1% CG intraperitoneally and 70 mg/kg TA. At the end of 21 days, all mice were killed by exsanguination under anesthesia with inhaled 5% isoflurane in room air and the parietal peritoneum was collected from each mouse for further experiments.

### Macrophage Culture

Raw264.7 cells were obtained from American Type Culture Collection and maintained in RPMI-1640 medium supplemented with 10% FBS, 1% penicillin and streptomycin in an atmosphere of 5% CO2 and 95% air at 37°C. We established two cell injured models in Raw264.7 cells in this study. (1) IL-4-stimulated model: IL-4 (10 ng/ml) was used to stimulate the transformation of Raw264.7 murine macrophage into M2 macrophage. Raw264.7 cells were starved for 12 hours and then exposed to IL-4 (10 ng/ml) for 12 hours in the presence or absence of different doses of TA (1µM, 5µM, 10µM) before cell harvesting. (2) HG-PDF-stimulated model: Different peritoneal dialysis fluid (HG-PDF, containing 1.5%, 2.5%, 4.25% glucose) was mixed with the cell culture medium (1:1 mix), then was used to stimulate the transformation of Raw264.7 murine macrophage into M2 macrophage. Raw264.7 cells were starved for 12 hours and then exposed to the HG-PDF for 36 hours in the presence or absence of different doses of TA (1µM, 5µM, 10µM) before cell harvesting. All of the *in vitro* experiments were repeated at least three times.

### Immunoblot Analysis

Cell samples and peritoneal tissue samples were prepared and determined total protein concentration (μg/μl) for clarified homogenates by bicinchoninic acid (BCA) assay, according to the manufacturer’s instructions (ThermoFisher). Proteins were separated by SDS-PAGE Gel electrophoresis (8%-12%) in 120V for 90 minutes and transferred to 0.2 mm nitrocellulose membranes (80V for 80 minutes). After incubation with 5% nonfat milk for 1 hour at room temperature, the membranes were incubated with primary antibodies overnight at 4°C and then incubated with appropriate horseradish peroxidase-conjugated secondary antibodies for 1 hour on the shaker at room temperature. Membranes were washed three times with prewarmed TBST in 10 minutes. Bound antibodies were visualized by chemiluminescence detection. Densitometry analysis of immunoblot results was conducted by using Image J software.

### Morphologic Studies of Peritoneum

The fixed peritoneum tissues were embedded in paraffin, cut into 3-μm-thick sections, and sectioned onto slides. The slides were stained with Masson’s trichrome staining and Sirius red staining to evaluate the degree of fibrosis and collagen deposits. The staining was performed according to the protocol provided by the supplier (Sigma-Aldrich). The positive area of Masson’s trichrome staining and Sirius red staining were quantitatively measured using Image Pro-Plus software (Media-Cybernetics, Silver Spring, MD, USA) by drawing a line around the perimeter of positive staining area, and the ratio to each microscopic field was calculated and graphed. Slides were captured with a Nikon Eclipse 80i microscope equipped with a digital camera (DS-Ri1, Nikon, Shanghai, China).

### Immunohistochemical and Immunofluorescence Staining

Immunohistochemical and immunofluorescence staining were carried out according to the procedure described in our previous study (32, 33). FFPE sections (3 μm) were rehydrated and incubated with primary antibodies against α-SMA (1:100, ab5694, Abcam), Twist (1:100, ab175430, Abcam), HDAC6 (1:100, A11259, ABclonal), CD163 (1:100, GB11340-1, Servicebio), Collagen I (1:400, GB11022-3, Servicebio), CD68 (1:100, sc-20060, Santa Cruz Biotechnology) and Arginase-1 (1:100, #93668, Cell Signaling Technology), and then secondary antibodies (Invitrogen). Slides were captured with a Nikon Eclipse 80i microscope equipped with a digital camera (DS-Ri1, Nikon, Shanghai, China).

### Statistical Analysis

All the experiments were conducted at least three times. Data depicted in graphs are expressed as means ± S.E.M. for each group. Student’s t-test was employed for comparisons between two groups and one-way analysis of variance (ANOVA) followed by Tukey’s post-test for multiple comparisons was used for groups of three or more. All tests were two-tailed. The *p*-value less than 0.05 was considered statistically significant and was marked in each graph. P<0.05 was considered significant. The statistical analyses were conducted by using IBM SPSS Statistics 20.0 (Version X; IBM, Armonk, NY, USA).

## Results

### Administration of TA Effectively Suppresses HDAC6 Expression and CG-Induced Peritoneal Fibrosis

**Figure 1:**
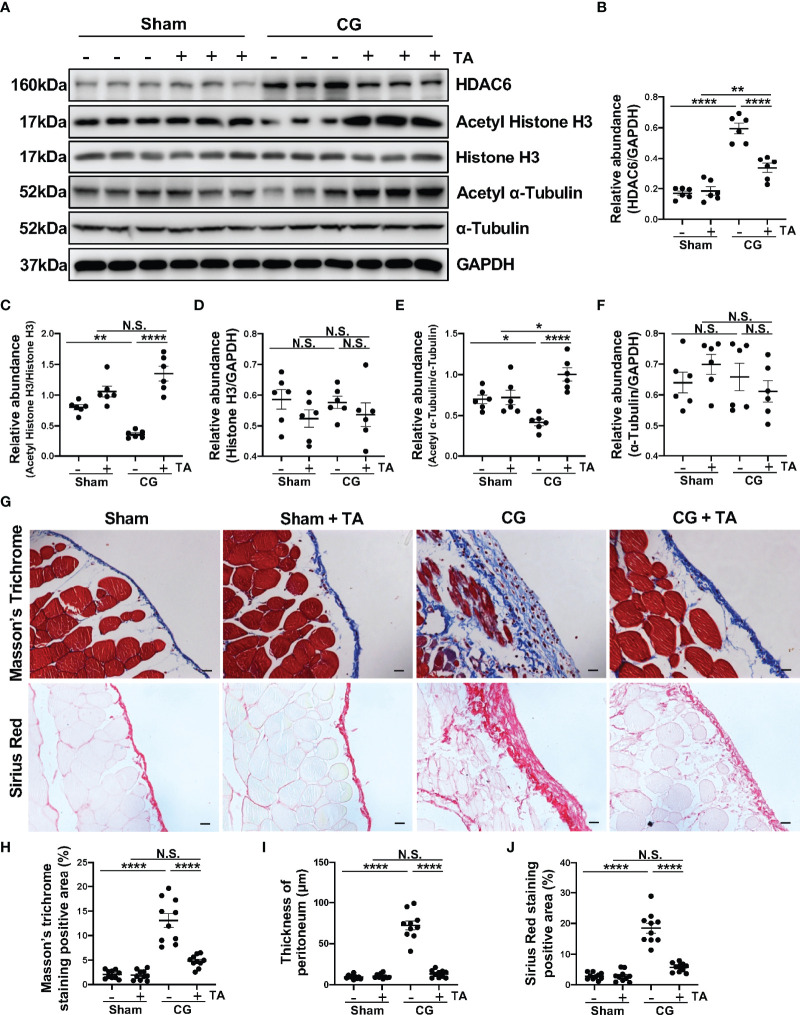
TA effectively suppresses HDAC6 expression and CG-induced peritoneal fibrosis. **(A)** Western blot analysis showed the protein levels of HDAC6, Acetyl Histone H3, Histone H3, Acetyl α-Tubulin, α-Tubulin, and GAPDH in peritoneum from different groups of mice. Expression levels of **(B)** HDAC6, **(C)** Acetyl Histone H3, **(D)** Histone H3, **(E)** Acetyl α-Tubulin, and **(F)** α-Tubulin in different groups were quantified by densitometry and normalized with GAPDH, Histone H3, and α-Tubulin respectively. **(G)** Representative micrographs of Masson’s Trichrome staining and Sirius Red staining of the peritoneum from different groups of mice. **(H)** The positive area of Masson’s Trichrome staining-positive submesothelial area (blue). **(I)** The thickness of peritoneum according to Masson’s Trichrome staining. **(J)** The positive area of Sirius Red-positive submesothelial area (red). Data were expressed as means ± SEM. *P<0.05, **P<0.01, ****P<0.0001. All scale bars = 20 μm. NS: P≥0.05.

To better clarify the role of HDAC6 in peritoneal fibrosis, we established a murine peritoneal fibrosis model by intraperitoneally injecting 0.1% CG every other day for 21 days. In addition, we used TA, a selective inhibitor of HDAC6, to explore the effects of HDAC6 inhibition in this study. In the group of mice that only accepted CG injection, we found a relatively higher expression of HDAC6 companied with the down-regulation of Acetyl Histone H3 and Acetyl α-Tubulin. This trend, however, could be effectively reversed by TA treatment (**Figures 1A–F**). The Masson’s Trichrome staining and Sirius Red staining showed the mice that accepted the treatment of TA had a slighter morphological change in peritoneum compared to their counterparts which only received intraperitoneal injection of CG (**Figure 1G**). We also performed the morphometric quantification of the positive area and thickness of the peritoneum (**Figures 1H–J**), further confirming the anti-fibrogenic effect of TA. All the data showed us that HDAC6 overexpressed in the CG-induced peritoneal fibrosis and the degree of peritoneal fibrosis injury could be ameliorated by inhibition of HDAC6.

### TA Inhibits CG-Induced EMT and ECM Protein Deposition in the Fibrotic Peritoneum

**Figure 2:**
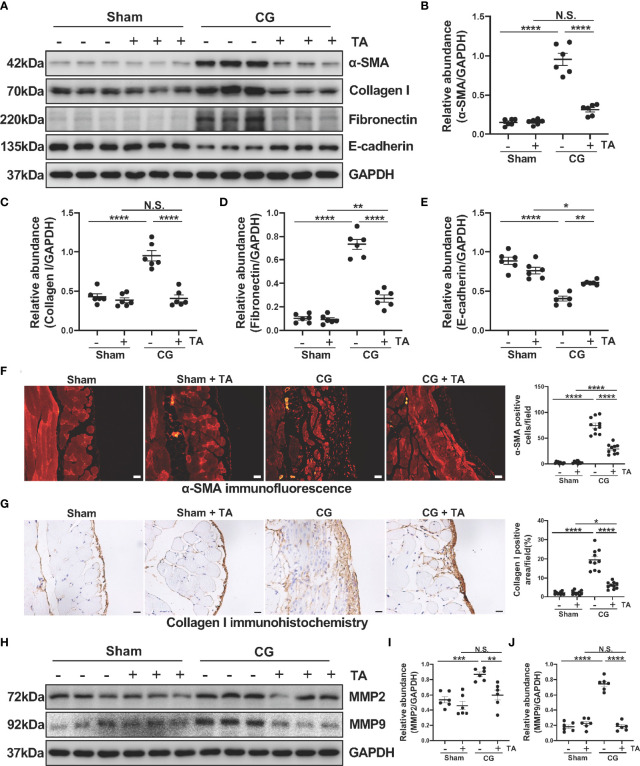
TA inhibits CG-induced EMT and ECM protein deposition in the fibrotic peritoneum. **(A)** Western blot analysis showed the protein levels of α-SMA, Collagen I, Fibronectin, E-cadherin, and GAPDH in peritoneum from different groups of mice. Expression levels of **(B)** α-SMA, **(C)** Collagen I, **(D)** Fibronectin, and **(E)** E-cadherin in different groups were quantified by densitometry and normalized with GAPDH. **(F)** Representative micrographs of immunofluorescence staining of α-SMA and quantization count of α-SMA-positive cells. **(G)** Representative micrographs of immunohistochemical staining of Collagen I and quantization of Collagen I-positive area (%). **(H)** Western blot analysis showed the protein levels of MMP2, MMP9, and GAPDH in peritoneum from different groups of mice. Expression levels of **(I)** MMP2 and **(J)** MMP9 in different groups were quantified by densitometry and normalized with GAPDH. Data were expressed as means ± SEM. *P<0.05, **P<0.01, ***P<0.001, ****P<0.0001. All scale bars = 20 μm. NS: P≥0.05.

To further explore the role of HDAC6 in the process of EMT and ECM protein deposition, we analyzed protein levels of α-SMA, Collagen I, Fibronectin and E-cadherin in the mice peritoneum. We observed elevated levels of α-SMA, Collagen I, Fibronectin, and decreased level of E-cadherin in the CG-injected group, while TA treatment effectively up-regulated the E-cadherin expression and down-regulated several ECM proteins, including Collagen I and Fibronectin (**Figures 2A–E**). To confirm this observation, we next conducted immunofluorescence staining for α-SMA and immunohistochemical staining for Collagen I. The results (**Figures 2F, G**) also demonstrated that TA prominently decreased α-SMA and Collagen I in CG-injured peritoneum tissues. Several previous studies have reported that MMP2 and MMP9, two members of matrix metalloproteinases, are linked to EMT and ECM protein deposition (34, 35). The inhibition of the MMP2 was reported to decrease cardiac fibrosis (36). So, we detected the expression levels of MMP2 and MMP9 in the mice’s peritoneum. After comparing the protein levels in different groups, we found that administration of TA reduced the MMP2 and MMP9 levels in CG-induced fibrotic peritoneum (**Figures 2H–J**). Collectively, these results showed that inhibition of HDAC6 by TA could block the EMT process of peritoneal mesothelial cells and ECM protein deposition within the peritoneum.

### Inhibition of HDAC6 Prevents Macrophage Infiltration and Alternatively Activated Macrophages (M2) Polarization in CG-Induced Mouse Model

**Figure 3:**
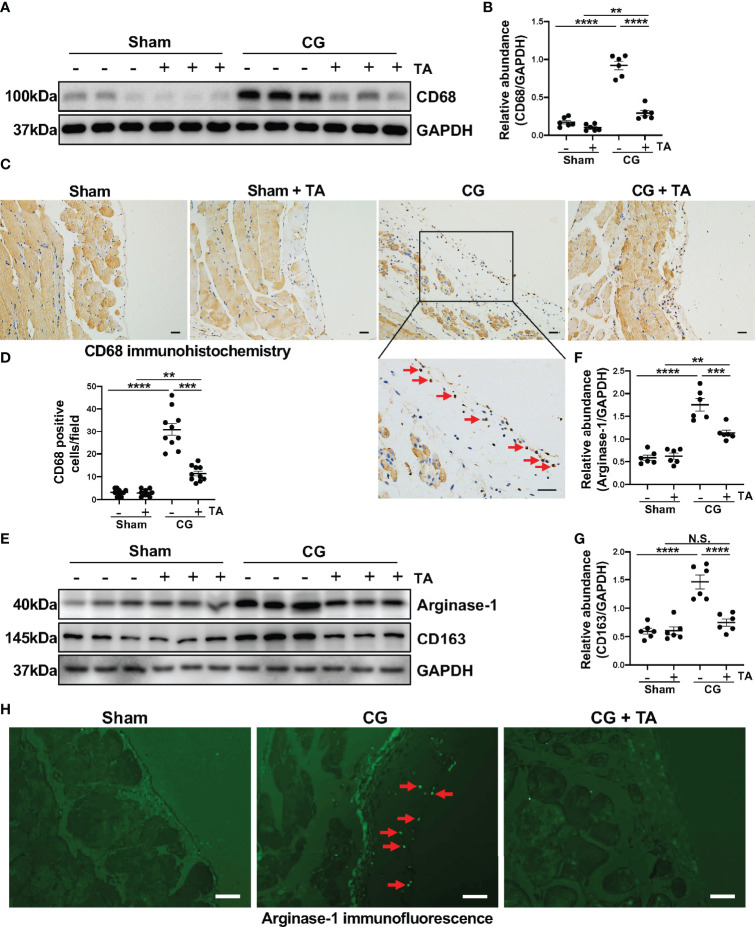
Inhibition of HDAC6 prevents macrophage infiltration and alternatively activated macrophages (M2) polarization in CG-induced mouse model. **(A)** Western blot analysis showed the levels of CD68 and GAPDH in peritoneum from different groups of mice. **(B)** Expression levels of CD68 in different groups were quantified by densitometry and normalized with GAPDH. **(C)** Representative micrographs of immunohistochemical staining of CD68 on peritoneum from different groups of mice. Red arrows represent CD68 positive cells. **(D)** Quantization count of CD68-positive cells. **(E)** Western blot analysis showed the levels of Arginase-1, CD163, and GAPDH in peritoneum from different groups of mice. Expression levels of **(F)** Arginase-1 and **(G)** CD163 in different groups were quantified by densitometry and normalized with GAPDH. **(H)** Representative micrographs of immunofluorescence staining of Arginase-1 in peritoneum from different groups of mice. Red arrows represent Arginase-1 positive cells. Data were expressed as means ± SEM. **P<0.01, ***P<0.001, ****P<0.0001. All scale bars = 20 μm. NS: P≥0.05.

Considering the importance of macrophage in peritoneal fibrosis, we further observed the infiltration and polarization of macrophage in a mouse peritoneal fibrosis model established by CG. The results of immunoblotting and immunohistochemistry suggested that macrophages infiltrated heavily in the fibrotic peritoneal tissue with the increased expression level of CD68 (the cell marker of macrophages) (**Figures 3A–D**). TA treatment significantly down-regulated the expression of CD68 and reduced CD68-positive cells in the thickened peritoneum (**Figures 3A–D**). The enhanced M2 macrophage polarization has been taken as the pro-fibrotic factor in various fibrotic diseases, including peritoneal fibrosis. To assess whether the inhibition of HDAC6 by TA affected the M2 polarization, we again analyzed the levels of characteristic markers of M2 macrophages in peritoneum. The Arginase-1 [a functional marker of M2 phenotype (37)] and CD163 [a cell-surface marker of M2 phenotype (37)] expressed relatively higher in the CG group, while TA treatment could effectively decrease the expression of Arginase-1 and CD163 (**Figures 3E–G**). The immunofluorescence staining of Arginase-1 also showed that TA could reduce the Arginase-1 positive cells in peritoneum after CG injection (**Figure 3H**). Taken together, these results suggested that M2 macrophage polarization was involved in the process of peritoneal fibrosis, and inhibition of HDAC6 by TA could not only prevent the macrophage infiltration but also M2 polarization, resulting in amelioration of peritoneal fibrosis.

### Inhibition of HDAC6 by TA Prevents M2 Macrophage Polarization *via* Suppressing TGF-β/Smad3 Signaling Pathway *In Vivo*

**Figure 4:**
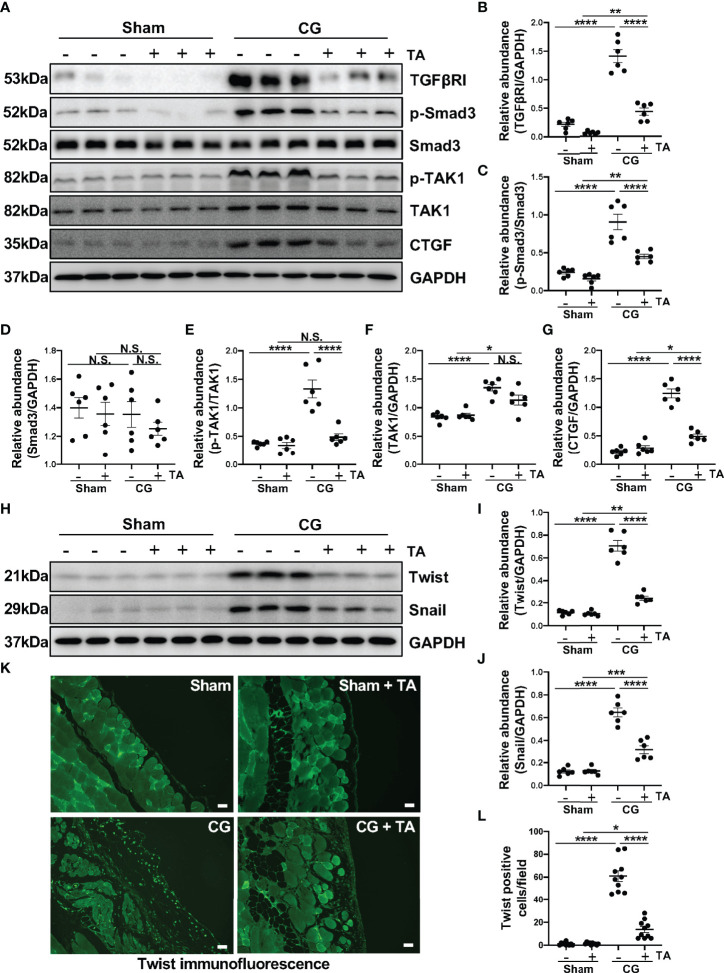
Inhibition of HDAC6 by TA prevents M2 macrophage polarization *via* suppressing TGF-β1/Smad3 signaling *in vivo*. **(A)** Western blot analysis showed the levels of TGFβRI, p-Smad3, Smad3, p-TAK1, TAK1, CTGF, and GAPDH in peritoneum from different groups of mice. Expression levels of **(B)** TGFβRI, **(C)** p-Smad3, **(D)** Smad3, **(E)** p-TAK1, **(F)** TAK1, and **(G)** CTGF in different groups were quantified by densitometry and normalized with GAPDH, Smad3, TAK1 respectively. **(H)** Western blot analysis showed the levels of Twist, Snail, and GAPDH in peritoneum from different groups of mice. Expression levels of **(I)** Twist and **(J)** Snail in different groups were quantified by densitometry and normalized with GAPDH. **(K)** Representative micrographs of immunofluorescence staining of Twist on peritoneum from different groups of mice. **(L)** Quantization count of Twist-positive cells. Data were expressed as means ± SEM. *P<0.05, **P<0.01, ****P<0.0001. All scale bars = 20 μm. NS: P≥0.05.

TGF-β1/Smad3 signaling pathway is proved to be critical in the pathology studies of peritoneal fibrosis (38, 39), and recent research reports that exposure to TGF-β1 can induce the M2 polarization in macrophages (40). We wondered whether the inhibition of HDAC6 manipulated the TGF-β1/Smad3 in M2 macrophage polarization. In this study, the intraperitoneal injection of CG induced upregulation of TGFβRI and connective tissue growth factor [CTGF, an important downstream mediator of TGF-β1 in fibrotic process (41)], as well as promoted the phosphorylation of Smad3 and TAK1 (**Figures 4A–G**). In the treatment group, TA could inhibit TGF-β1/Smad3 signaling pathway and its downstream signal, TAK1 and CTGF (**Figures 4A–G**). We further detected the expressions of Twist and Snail, which are two key nuclear transcription factors of TGF-β1 signal (42). Both of them were almost not expressed in the sham group with/without TA injection, while CG prolonged exposure markedly increased their expressions in fibrotic peritoneal tissue. TA treatment had the ability to reduce expression levels of Twist and Snail, and decrease the Twist positive cells in immunofluorescence staining (**Figures 4H–L**). Therefore, TA could inhibit TGF-β1/Smad3 signaling pathway, and then prevent M2 macrophage polarization in peritoneal fibrosis.

### Inhibition of HDAC6 by TA Prevents M2 Macrophage Polarization *via* Suppressing PI3K/AKT, STAT3 and STAT6 Signaling *In Vivo*

**Figure 5:**
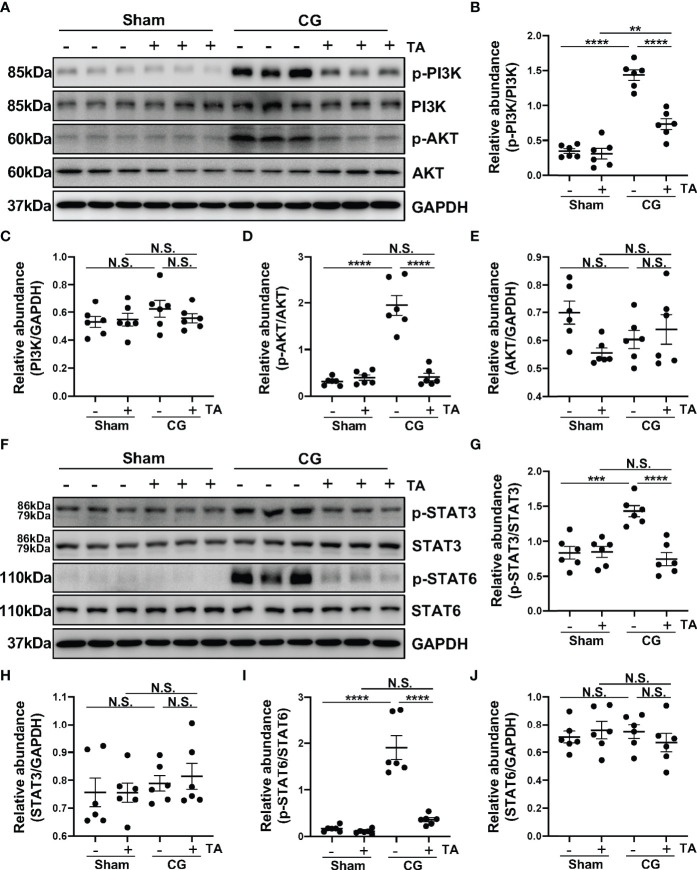
Inhibition of HDAC6 by TA prevents M2 macrophage polarization *via* suppressing PI3K/AKT, STAT3 and STAT6 signaling *in vivo*. **(A)** Western blot analysis showed the levels of p-PI3K, PI3K, p-AKT, AKT and GAPDH in peritoneum from different groups of mice. Expression levels of **(B)** p-PI3K, **(C)** PI3K, **(D)** p-AKT, and **(E)** AKT in different groups were quantified by densitometry and normalized with GAPDH, PI3K, AKT respectively. **(F)** Western blot analysis showed the levels of p-STAT3, STAT3, p-STAT6, STAT6, and GAPDH in peritoneum from different groups of mice. Expression levels of **(G)** p-STAT3, **(H)** STAT3, **(I)** p-STAT6, and **(J)** STAT6 in different groups were quantified by densitometry and normalized with GAPDH, STAT3, and STAT6 respectively. Data were expressed as means ± SEM. **P<0.01, ***P<0.001, ****P<0.0001. NS: P≥0.05.

Phosphatidylinositol 3-kinase (PI3K)/protein kinase B (PKB/AKT) signaling pathway is one of the important signaling pathways manipulating proliferation, metabolism, and survival. In pulmonary fibrosis, AKT can induce M2 macrophages to produce pro-fibrotic cytokines promoting fibrosis (43). Furthermore, PI3K activation is supposed to enhance M2 polarization in bleomycin lung fibrosis (44). To determine whether HDAC6 regulated M2 polarization process through PI3K/AKT signaling pathway, we detected levels of p-PI3K, PI3K, p-AKT, and AKT in the peritoneum from different groups. CG-injection in mice up-regulated the phosphorylation of PI3K and AKT, compared with the sham group, while TA treatment could decrease the ratio of p-PI3K/PI3K and p-AKT/AKT (**Figures 5A–E**). This meant inhibition of HDAC6 by TA blocked the activation of PI3K/AKT signaling pathway. Numerous studies have suggested that the activated STAT6 and STAT3 enhance the M2 polarization and suppress the M1 polarization (45–47). According to our results, the phosphorylation of STAT3 and STAT6 was increased in the peritoneum from mice in the CG group, and the rise of phosphorylation was significantly suppressed by the administration of TA (**Figures 5F–J**). These results suggested that inhibition of HDAC6 might suppress the M2 macrophage polarization by regulating the PI3K/AKT, STAT3 and STAT6 signaling pathways.

### TA Inhibits M2 Macrophage Polarization in Both IL-4 and HG-PDF Stimulated Raw264.7 Cells

**Figure 6:**
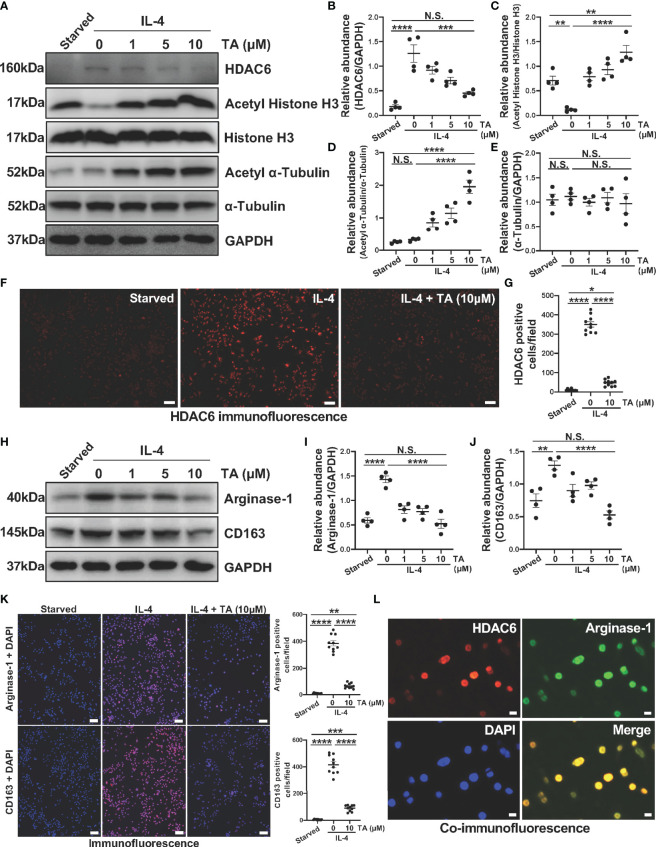
TA inhibits M2 macrophage polarization in IL-4-stimulated Raw264.7 cells. **(A)** Western blot analysis showed the levels of HDAC6, Acetyl Histone H3, Histone H3, Acetyl α-Tubulin, α-Tubulin, and GAPDH in IL-4-stimulated Raw264.7 cells treated with different doses of TA. Expression levels of **(B)** HDAC6, **(C)** Acetyl Histone H3, **(D)** Acetyl α-Tubulin, and **(E)** α-Tubulin in different groups were quantified by densitometry and normalized with GAPDH, Histone H3, and α-Tubulin respectively. **(F)** Representative micrographs of immunofluorescence staining of HDAC6 in Raw264.7 cells with different treatments (Scale bars = 50 μm). **(G)** Quantization count of HDAC6-positive cells was calculated. **(H)** Western blot analysis showed the levels of Arginase-1, CD163, and GAPDH in IL-4-stimulated Raw264.7 cells treated with different doses of TA. Expression levels of **(I)** Arginase-1, and **(J)** CD163 in different groups were quantified by densitometry and normalized with GAPDH. **(K)** Representative micrographs of immunofluorescence staining of Arginase-1 and CD163 in Raw264.7 cells with different treatments, and quantization count of Arginase-1 and CD163 positive cells (Scale bars = 50 μm). **(L)** Co-immunofluorescence staining of HDAC6 (red) and Arginase-1 (green) in the Raw264.7 cells (Scale bars = 10 μm). Data were expressed as means ± SEM. *P<0.05, **P<0.01, ***P<0.001, ****P<0.0001. NS: P≥0.05.

The above results have clarified the relationship between HDAC6 and macrophage polarization in CG-associated peritoneal fibrosis mouse model, then we further explored the role and mechanism of HDAC6-mediated macrophage polarization in Raw264.7 cells. IL-4 was a well-known stimulator to induce M2 macrophage polarization. Compared to the starved cells, HDAC6 overexpressed in the Raw264.7 cells stimulated by IL-4 (**Figures 6A, B**). TA was able to suppress the expression of HDAC6, and up-regulate the Acetyl Histone H3 and Acetyl α-Tubulin in a dose-dependent way (**Figures 6A–E**). However, TA had no influence on total Histone H3 and total α-Tubulin protein levels. The immunofluorescence staining for HDAC6 showed the portion of HDAC6 positive cells reduced, confirming the effective inhibition of HDAC6 in the IL-4-stimulated Raw264.7 cells by TA (**Figures 6F, G**). Furthermore, IL-4 stimulation increased Arginase-1 and CD163 expressions, implying the polarization of Raw264.7 to M2. TA treatment down-regulated the expression of Arginase-1 and CD163 from immunoblot analysis and immunofluorescence staining (**Figures 6H–K**). In addition, the co-staining of HDAC6 and Arginase-1 indicated the involvement of HDAC6 in IL-4-induced M2 polarization (**Figure 6L**).

**Figure 7:**
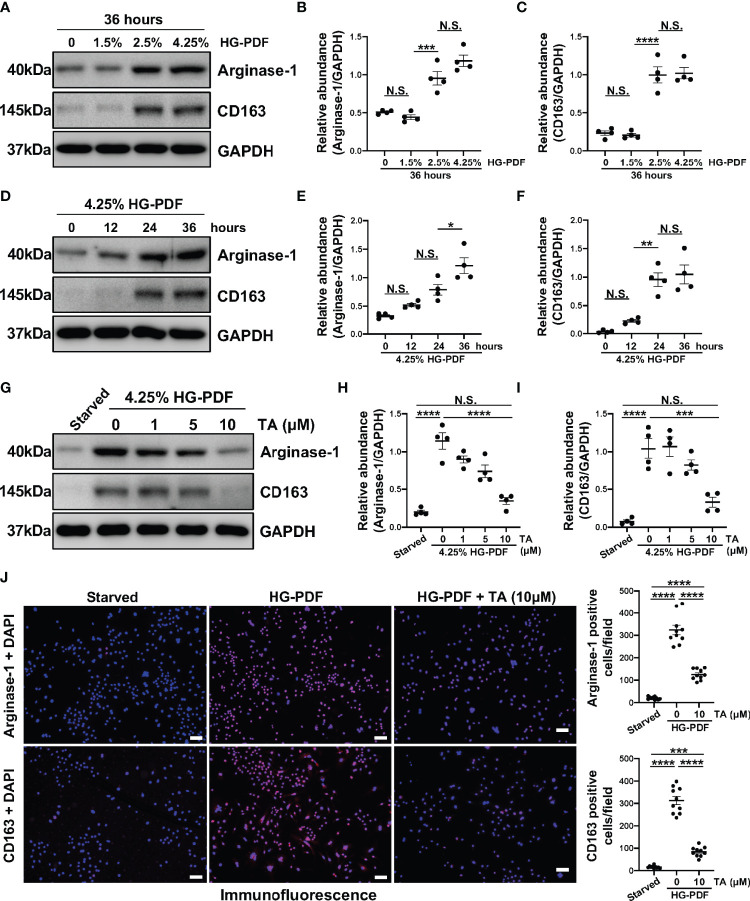
TA inhibits M2 macrophage polarization in HG-PDF stimulated Raw264.7 cells. **(A)** Western blot analysis showed the levels of Arginase-1, CD163, and GAPDH in Raw264.7 cells treated with peritoneal dialysis fluid in different dextrose concentrations. Expression levels of **(B)** Arginase-1 and **(C)** CD163 in different groups were quantified by densitometry and normalized with GAPDH. **(D)** Western blot analysis showed the levels of Arginase-1, CD163, and GAPDH in Raw264.7 cells treated with 4.25% HG-PDF for different times. Expression levels of **(E)** Arginase-1 and **(F)** CD163 in different groups were quantified by densitometry and normalized with GAPDH. **(G)** Western blot analysis showed the levels of Arginase-1, CD163, and GAPDH in 4.25% HG-PDF treated Raw264.7 cells with different doses of TA. Expression levels of **(H)** Arginase-1 and **(I)** CD163 in different groups were quantified by densitometry and normalized with GAPDH. **(J)** Representative micrographs of immunofluorescence staining of Arginase-1 and CD163 in Raw264.7 cells with different treatments. Quantization count of Arginase-1 and CD163 positive cells was calculated. Data were expressed as means ± SEM. *P<0.05, **P<0.01, ***P<0.001, ****P<0.0001. All scale bars = 50 μm. NS: P≥0.05.

On the other hand, high glucose has been proved to mediate the polarization of peritoneal macrophages to the M2 phenotype (48). So, we further developed another *in vitro* model in Raw264.7 cells treated with different peritoneal dialysis fluid (HG-PDF, containing 1.5%, 2.5%, 4.25% glucose). Immunoblotting results revealed that HG-PDF was able to induce M2 macrophage polarization with increased expressions of Arginase-1 and CD163 in both dose and time dependent (**Figures 7A–F**). Then we similarly treated the Raw264.7 cells with different doses of TA after stimulating by 4.25% HG-PDF. The TA at a concentration of 10μM successfully decreased the expression levels of Arginase-1 and CD163 as expected (**Figures 7G–I**). The immunofluorescence staining further determined the suppression of M2 polarization by TA (**Figure 7J**). All these results demonstrated that TA could inhibit M2 macrophage polarization in both IL-4 and HG-PDF stimulated Raw264.7 cells.

### TA Inhibits M2 Macrophage Polarization *via* Suppressing TGF-β1/Smad3, PI3K/AKT, STAT3 and STAT6 Signaling in Raw264.7 Cells Stimulated by IL-4 and HG-PDF

**Figure 8:**
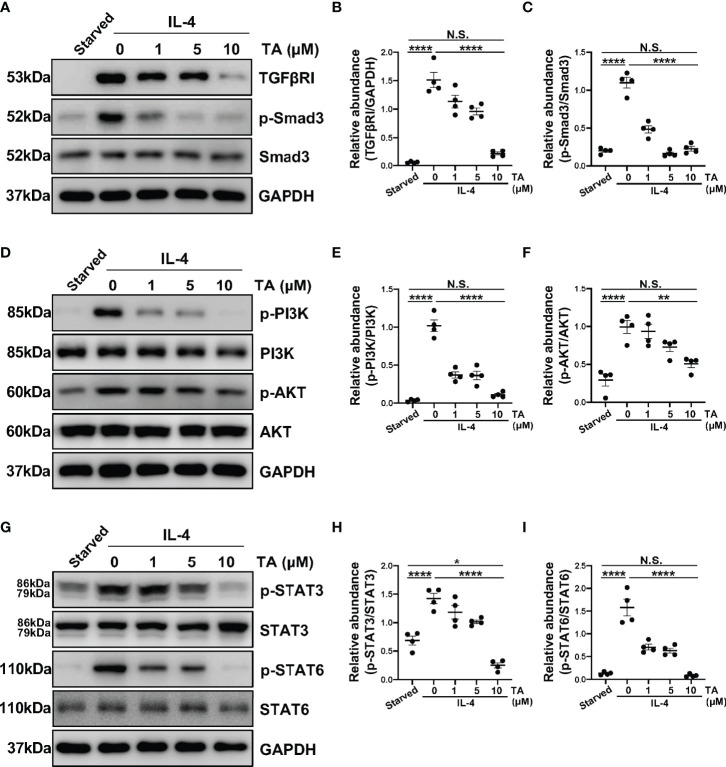
TA inhibits M2 macrophage polarization *via* suppressing TGF-β1/Smad3, PI3K/AKT, STAT3 and STAT6 signaling in Raw264.7 cells stimulated by IL-4. **(A)** Western blot analysis showed the levels of TGFβRI, p-Smad3, Smad3, and GAPDH in IL-4-stimulated Raw264.7 cells treated with different doses of TA. Expression levels of **(B)** TGFβRI and **(C)** p-Smad3 in different groups were quantified by densitometry and normalized with GAPDH and Smad3 respectively. **(D)** Western blot analysis showed the levels of p-PI3K, PI3K, p-AKT, AKT and GAPDH in IL-4-stimulated Raw264.7 cells treated with different doses of TA. Expression levels of **(E)** p-PI3K and **(F)** p-AKT in different groups were quantified by densitometry and normalized with PI3K and AKT respectively. **(G)** Western blot analysis showed the levels of p-STAT3, STAT3, p-STAT6, STAT6, and GAPDH in IL-4-stimulated Raw264.7 cells treated with different doses of TA. Expression levels of **(H)** p-STAT3 and **(I)** p-STAT6 in different groups were quantified by densitometry and normalized with STAT3 and STAT6 respectively. Data were expressed as means ± SEM. *P<0.05, **P<0.01. NS: P≥0.05.

**Figure 9:**
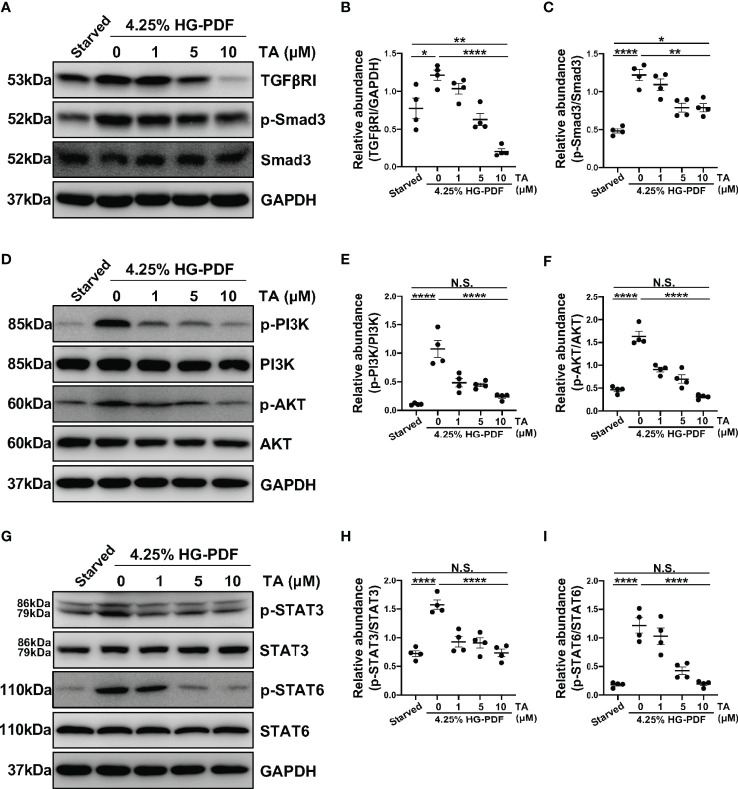
TA inhibits M2 macrophage polarization *via* suppressing TGF-β1/Smad3, PI3K/AKT, STAT3 and STAT6 signaling in Raw264.7 cells stimulated by HG-PDF. **(A)** Western blot analysis showed the levels of TGFβRI, p-Smad3, Smad3, and GAPDH in 4.25% HG-PDF treated Raw264.7 cells with different doses of TA. Expression levels of **(B)** TGFβRI and **(C)** p-Smad3 in different groups were quantified by densitometry and normalized with GAPDH and Smad3 respectively. **(D)** Western blot analysis showed the levels of p-PI3K, PI3K, p-AKT, AKT, and GAPDH in 4.25% HG-PDF treated Raw264.7 cells with different doses of TA. Expression levels of **(E)** p-PI3K and **(F)** p-AKT in different groups were quantified by densitometry and normalized with PI3K and AKT respectively. **(G)** Western blot analysis showed the levels of p-STAT3, STAT3, p-STAT6, STAT6, and GAPDH in 4.25% HG-PDF treated Raw264.7 cells with different doses of TA. Expression levels of **(H)** p-STAT3 and **(I)** p-STAT6 in different groups were quantified by densitometry and normalized with STAT3 and STAT6 respectively. Data were expressed as means ± SEM. *P<0.05, **P<0.01, ****P<0.0001. NS: P≥0.05.

*In vitro*, we also detected several signaling pathways that HDAC6 contributed to M2 macrophage polarization. As expected, the expression of TGFβRI and p-Smad3 rose in the IL-4-stimulated Raw264.7 cells (**Figures 8A–C**). Consistently, the administration of TA could suppress the expression of TGFβRI and p-Smad3 in a dose-dependent manner, suggesting that TA inactivated TGF-β1/Smad3 signaling after IL-4 stimulation (**Figures 8A–C**). Moreover, IL-4 stimulation could activate PI3K/AKT, STAT3 and STAT6 signaling pathways, while TA treatment dose-dependently inhibited all of them, especially in the dose of 10μM (**Figures 8D–I**). It was noteworthy that neither IL-4 stimulation nor TA treatment could influence the total protein of Smad3, PI3K, AKT, STAT3 or STAT6. Similarly, inhibition of HDAC6 with TA also blocked these aforementioned signaling pathways in 4.25% HG-PDF-stimulated Raw264.7 cells (**Figure 9**). In conclusion, TA inhibited both IL-4 and HG-PDF induced alternatively activated macrophages (M2) polarization in Raw264.7 cells *via* suppression of TGF-β1/Smad3, PI3K/AKT, STAT3 and STAT6 signaling pathways.

## Discussion

HDAC6 is a class IIb member of the HDAC family and takes α-tubulin as deacetylation modification substrate protein by interactions with microtubules (24, 25). The inhibition of HDAC6 is reported to exert the anti-fibrotic effect in various fibrosis models (26, 27, 49). Recently we have demonstrated that HDAC6 is critically implicated in HG-PDF induced peritoneal fibrosis, and overexpressed in the peritoneum and dialysis effluent from PD patients (29). However, the precise mechanisms of HDAC6 in peritoneal fibrosis have not been elucidated. In this study, we further proved that HDAC6 could aggravate CG-induced peritoneal fibrosis *via* promoting macrophage polarization to the M2 phenotype. Moreover, our results revealed that blockade of HDAC6 inhibited alternatively M2 macrophages polarization by suppressing the activation of TGF-β/Smad3, PI3K/AKT, and STAT3, STAT6 pathways. Therefore, this study clarified the importance of HDAC6 in peritoneal fibrosis from a new mechanism point that HDAC6 contributed to M2 macrophage polarization.

To confirm the therapeutic effect of HDAC6 inhibition, we treated the CG-induced peritoneal fibrosis mouse model with TA and found a significant alleviation of fibrosis and reduced extracellular matrix protein deposition. These changes are symbols of remission of epithelial-mesenchymal transition, a key factor in fibrogenesis (50). So far, a great bulk of evidence has elucidated the role of macrophage polarization, especially M2 polarization, in the pathological progression of fibrosis (40, 51, 52). The triggered M2 macrophage polarization promotes EMT and metastasis in gastric cancer (53). Clinical data showed the infiltration of CD163 positive macrophages associated with EMT in colorectal cancer metastasis (54). The latest paper demonstrated that the M2c (a subtype of M2 macrophages) macrophage polarization could enhance the EMT of peritoneal mesothelial cells (55). It is consistent with our observation in the mouse model: the expression of Arginase-1 and CD163 elevated in the peritoneum tissues from the CG-injected group. We confirmed the increased infiltration of macrophages and enhanced M2 polarization in the peritoneal fibrosis. In addition, the staining colocalized HDAC6 and Arginase-1 in IL-4-stimulated Raw264.7 cells, which provided support that HDAC6 might promote peritoneal fibrosis *via* manipulating M2 polarization. These results pointed out that the therapeutic effect of peritoneal fibrosis by TA correlated with M2 polarization, and encouraged us to explore the further mechanisms.

It is well established that the TGF-β signaling could regulate macrophage behavior and polarize the macrophages towards a certain phenotype (56). TGF-β/Smad3 signaling was found to stimulate macrophages to synthesize pro-inflammatory cytokines. TGF-β could activate Smad3 in macrophages, and the TGF-β/Smad3 activation exerted protective effects by stimulating phagocytosis of macrophages in myocardial infarction (57). In research of chronic renal allograft rejection, the increased M2 macrophages and elevated nuclear-phosphorylated Smad3 contributed to the interstitial fibrosis, suggesting enhanced M2 polarization *via* a Smad3-dependent mechanism in the development of interstitial fibrosis (58). Considering the role of TGF-β/Smad3 signaling in macrophage polarization, we also detected the regulation of TA on TGF-β/Smad3 during peritoneal fibrosis in this study. The activation of TGF-β/Smad3 signaling was observed in peritoneum tissues from CG-injected mice and in two cell injured models, and this activation was considerably suppressed by TA administration. Besides, TA also blocked downstream signaling molecules of TGF-β/Smad3, including TAK1 and CTGF, and two related nuclear transcription factors. Our results support that the HDAC6 could enhance M2 polarization to promote peritoneal fibrosis *via* regulating the TGF-β/Smad3 signaling. However, the macrophage-specific Smad3 loss in the previous study could not affect the angiogenesis and fibrogenesis significantly (57), suggesting us other mechanisms might involve in the manipulation of M2 polarization.

Thus, we next demonstrated whether the phosphatidylinositol 3-kinase/protein kinase B (PI3K/AKT) signaling pathway participated in the regulation of macrophage polarization by HDAC6. Based on the previous literature, the PI3K/AKT pathway affected survival, migration, metabolic progress, and polarization in macrophages (20). The PI3K/AKT pathway was found to promote M2 polarization in neuroinflammation (59, 60). Cao et al. found that HDAC6 could control PI3K regulatory subunit 2 (PIK3R2) through miR-30d, thus activating PI3K/AKT/mTOR and ERK pathways (61). Another research showed that HDAC6 physically interacted with AKT and acetylated AKT at Lys163 and Lys377 located in the kinase domain. This research treated the deacetylase activity of HDAC6 as a novel regulator of AKT signaling (62). The above results suggest the involvement of HDAC6 activation in PI3K/AKT signaling pathway. In this study, we demonstrated that the activation of the PI3K/AKT pathway involved the effect of HDAC6 on M2 polarization. The inhibition of HDAC6 by TA effectively reduced the activation of the PI3K/AKT pathway *in vivo* and in Raw264.7 cells, indicating that HDAC6 regulated the PI3K/AKT pathway. Our findings were consistent with the previous study that activating the PI3K/AKT pathway promoted M2 polarization (63). In another word, the PI3K/AKT pathway mediated by HDAC6 was involved in the regulation of M2 polarization in peritoneal fibrosis.

The signal transducer and activator of transcription 3 also aroused our interest in exploring the mechanisms as the activity of HDAC1 and HDAC2 could negatively regulate STAT3 signaling (64). Furthermore, the activation of STAT3 was reported to induce macrophage differentiation toward the M2 phenotype (65, 66). The expression of p-STAT3 and p-STAT3/STAT3 levels in the peritoneum of our animal models and IL-4 or HG-PDF-treated macrophages were relatively higher. By contrast, the p-STAT3 and p-STAT3/STAT3 levels were reduced by TA administration. These data correspond to the findings by Yin, Z. et al.: the expression of STAT3 mRNA and protein was higher in IL-4-induced M2 macrophages (45). Our results originally implied the possible regulation of STAT3 by HDAC6. The underlying mechanism may be demonstrated by the suppression of IL-6/STAT3 signaling. Evidence has demonstrated that interfering with IL-6 trans-signaling could attenuate renal fibrosis *via* suppressing STAT3 activation while inhibiting the recruitment of macrophages (67). We previously observed elevation of IL-6 and macrophage infiltration in peritoneal fibrosis rats. Therefore, we conceived that there might be interactions between the inhibition of HDAC6 and the IL-6/STAT3 signaling, leading to a phenotypic switch in macrophages. However, the detailed relationship needs further elucidation.

In addition, we decided to determine the link between STAT6 and HDAC6 due to the interactions between HDACs and STAT transcription factors. In fact, a recent study demonstrated that IL-4-STAT6 signaling was HDAC3 dependent and repressed the LPS-induced inflammatory program of macrophages (68). STAT6 is the major factor during macrophage M2 polarization (69). The activation of STAT6 is known to drive M2 polarization (70). The acetylation of STAT6 would further inhibit M2 polarization by suppressing the transcriptional activity of STAT6 (69). Moreover, the IL-4/STAT6 activation could regulate liver fibrosis (17) and cardiac fibrosis progression (71) through targeting macrophages. Our study showed that TA suppressed the expression of STAT6 in CG-induced peritoneal fibrosis and injured Raw264.7 cells. Accordingly, the results suggested that the regulation of M2 polarization by HDAC6 might exert *via* the IL-4/STAT6 signaling.

According to reported literature, a handful of selected HDAC6 inhibitors have been undergoing clinical trials for tumors treatment. In the public database of clinical trials from U.S. National Library of Medicine, there are seven HDAC6-related clinical trials with three studies completed, including multiple HDAC6 inhibitors (ACY-241, KA2507, ACY-1215). Results from a phase Ib study showed the combination therapy of ACY-241 (Citarinostat) and Nivolumab in advanced non-small cell lung cancer, and suggested the combination might be feasible in these patients (72). In another multicentre phase 1b trial, ACY-1215 (Ricolinostat) enhanced efficacy of lenalidomide and dexamethasone in relapsed or refractory multiple myeloma. 21 (55% [95% CI 38-71]) of 38 patients presented an overall response in a preliminary assessment of antitumour activity after combined medication with ACY-1215 (73). Overall, several clinical trials have proved the effectiveness, security, applicability of selected HDAC6 inhibitors in clinical treatment, which provides opinions and possibilities of the application of HDAC6 inhibitors in peritoneal fibrosis associated with peritoneal dialysis in the future.

In summary, the present study revealed the fibrogenic role of HDAC6 in CG-induced peritoneal fibrosis, and the anti-fibrotic effect of TA mediated by suppressing macrophage M2 polarization. We innovatively demonstrated that the inhibition of HDAC6 by TA significantly suppressed M2 macrophage polarization by regulating the TGF-β/Smad, PI3K/AKT, STAT3 and STAT6 signaling pathways. These findings indicated that targeting HDAC6 might be a novel therapeutic strategy for peritoneal fibrosis.

## Data Availability Statement

The raw data supporting the conclusions of this article will be made available by the authors, without undue reservation.

## Ethics Statement 

The animal study was reviewed and approved by Institutional Animal Care and Use Committee at Tongji University (Shanghai, RP China).

## Author Contributions

NL participated in research design. YS, JL, HC, YH, LT, XZ, MT, ZL, and SC conducted experiments. YS, JL, AQ, and NL contributed new reagents or analytic tools. YS, JL, and NL performed data analysis. YS, JL, NL, and AQ wrote or contributed to the writing of the manuscript. All authors approved the final version of the manuscript.

## Funding

This study was supported by the National Nature Science Foundation of China grants (82070791, 81670690, 81470991 and 81200492 to NL), the Outstanding Leaders Training Program of Pudong Health Bureau of Shanghai (PWR12021-02 to NL), the Shanghai Scientific Committee of China (20ZR1445800 and 13PJ1406900 to NL), the Shanghai Health Bureau and Shanghai administration of traditional Chinese Medicine of China (ZHYY-ZXYJHZX-202114 to NL), the Project of Pudong Health Bureau of Shanghai (PW2021D-04 and PWZxk2017-05 to NL).

## Conflict of Interest

The authors declare that the research was conducted in the absence of any commercial or financial relationships that could be construed as a potential conflict of interest.

## Publisher’s Note

All claims expressed in this article are solely those of the authors and do not necessarily represent those of their affiliated organizations, or those of the publisher, the editors and the reviewers. Any product that may be evaluated in this article, or claim that may be made by its manufacturer, is not guaranteed or endorsed by the publisher.
